# Amyloid precursor protein-b facilitates cell adhesion during early development in zebrafish

**DOI:** 10.1038/s41598-020-66584-8

**Published:** 2020-06-23

**Authors:** Rakesh Kumar Banote, Jasmine Chebli, Tuğçe Munise Şatır, Gaurav K. Varshney, Rafael Camacho, Johan Ledin, Shawn M. Burgess, Alexandra Abramsson, Henrik Zetterberg

**Affiliations:** 1 Institute of Neuroscience and Physiology, Department of Psychiatry and Neurochemistry, The Sahlgrenska Academy, University of Gothenburg, S-41345 Gothenburg, Sweden; 2 Translational and Functional Genomics Branch, National Human Genome Research Institute, National Institutes of Health, Bethesda, Maryland 20892 USA; 3 Genes & Human Disease Program, Oklahoma Medical Research Foundation, Oklahoma City, OK 73104 USA; 4 Centre for Cellular Imaging, Core Facilities, The Sahlgrenska Academy, University of Gothenburg, Gothenburg, Sweden; 5 Department of Organismal Biology, Science for Life Laboratory, Uppsala University, Uppsala, Sweden; 6 Clinical Neurochemistry Laboratory, Sahlgrenska University Hospital, Mölndal, Sweden; 7 Department of Neurodegenerative Disease, UCL Institute of Neurology, Queen Square, London, WC1N3BG United Kingdom; 8 UK Dementia Research Institute, London, WC1N3BG United Kingdom; 9 Present Address: Cellectricon AB, Neongatan 4B, SE-431 53, Mölndal, Sweden

**Keywords:** CRISPR-Cas9 genome editing, Cell adhesion, Gastrulation, Alzheimer's disease

## Abstract

Understanding the biological function of amyloid beta (Aβ) precursor protein (APP) beyond its role in Alzheimer’s disease is emerging. Yet, its function during embryonic development is poorly understood. The zebrafish APP orthologue, Appb, is strongly expressed during early development but thus far has only been studied via morpholino-mediated knockdown. Zebrafish enables analysis of cellular processes in an ontogenic context, which is limited in many other vertebrates. We characterized zebrafish carrying a homozygous mutation that introduces a premature stop in exon 2 of the *appb* gene. We report that *appb* mutants are significantly smaller until 2 dpf and display perturbed enveloping layer (EVL) integrity and cell protrusions at the blastula stage. Moreover, *appb* mutants surviving beyond 48 hpf exhibited no behavioral defects at 6 dpf and developed into healthy and fertile adults. The expression of the *app* family member, *appa*, was also found to be altered in *appb* mutants. Taken together, we show that *appb* is involved in the initial development of zebrafish by supporting the integrity of the EVL, likely by mediating cell adhesion properties. The loss of Appb might then be compensated for by other *app* family members to maintain normal development.

## Introduction

The amyloid beta (Aβ) precursor protein (APP) is an integral membrane protein recognized for its role in Alzheimer’s disease (AD) pathogenesis. It is a single-pass glycosylated type I transmembrane protein that is processed by proteases into numerous extracellular and intracellular biologically active fragments^1^. Cleavage of APP by β- and γ-secretases generates Aβ peptide fragments that aggregate into extracellular plaques in the brain with AD^2–4^. Moreover, recent studies have uncovered that APP might also contribute to AD-related neurodegeneration in Aβ-independent ways^5,6^.

APP is ubiquitously expressed with especially strong expression in the central nervous system^7–9^. However, its basic biological role has been difficult to elucidate, likely due to redundancy between APP family members^10^. Nevertheless, studies suggest fundamental functions of APP in axonal transport, cell differentiation and proliferation, synaptic transmission, learning and memory, neuronal development, neurite outgrowth, as well as in cell adhesion^11^. Mice with a deletion of *App* are viable and fertile but show reduced body weight, grip strength and locomotor activity^12,13^. Similarly, single *Aplp1* and *Aplp2* knockouts also display mild phenotypes. However, *App*^−/−^/*Aplp2*^−/−^ or *Aplp2*^−/−^/*Aplp1*^−/−^ double knockouts and triple knockouts (*App*^−/−^/*Aplp1*^−/−^/*Aplp2*^−/−^) show severe abnormalities and lethality^14^.

Here, we have performed a detailed characterization of zebrafish with a homozygous mutation in *appb*, generated with CRISPR/Cas9 technology. As an animal model, zebrafish has several advantages over, *e.g*., rodents; its external development, in combination with optical transparency, makes it possible to study cellular processes during early development in relation to genetic perturbation in a way that would be very challenging in mice. We analyzed morphological and behavioral changes, as well as potential compensatory mechanisms of other *app* family members, in *appb* mutants. During the blastula period, when zebrafish embryos transition from 128-cell stage to 50% epiboly, we observed enveloping layer (EVL) adhesion defects, of which the most severe were lethal. Our data suggest that this is caused by defects in cell adhesion. Similar to mice, mutant zebrafish were slightly smaller and did not show any behavioral alterations at an early age. However, loss of *appb* led to increased *appa* expression, as determined by analysis of both mRNA and protein expression. Overall, these results suggest early and partially lethal abnormalities in the development of *appb* mutants, which may be prevented by increased expression of other *app* family members.

## Materials and methods

### Animal care and ethics statement

Fish were maintained in stand-alone racks on a 14h:10h light:dark cycle at 28.5 °C, at the facility of the Institute of Neuroscience and Physiology, University of Gothenburg. System water was kept at a pH of 7.2–7.5 and conductivity of 600μS. Larvae were raised in embryo medium (EM) (1.0 mM MgSO_4_, 0.15 mM KH_2_PO_4,_ 0.042 mM Na_2_HPO_4,_ 1 mM CaCl_2,_ 0.5 mM KCl, 15 mM NaCl, 0.7 mM NaHCO_3_) at 28.5 °C in dark except for those in behavior tests that were maintained at a 14 h:10 h light:dark cycle. This study was approved by the Animal ethical committee in Gothenburg. All procedures for the experiments were performed in accordance with the animal welfare guidelines of the Swedish National Board for Laboratory Animals.

### Mutagenesis using the CRISPR/Cas9 system

The CRISPR/Cas9 system was used to generate zebrafish mutants, as previously described^15^. Briefly, gRNA synthesis was made with a cloning-free, oligo-based method using a target-specific DNA oligo (5′-GGATGACTCGGTGGGCTTGT-3′) and a ‘generic’ DNA oligo for the guide RNA. The two oligos were annealed and extended with DNA polymerase, and the resulting product serves as a template for *in vitro* transcription. Embryos were co-injected with 50 pg gRNA and 150 pg Cas9 mRNA, transcribed from the XbaI-linearized pT3TS-nls-zCas9-nls into the yolk at the one cell–stage. Injected embryos were screened for gRNA activity using a three-primer fluorescence PCR method^15^. A 260 base pair (bp) region surrounding the target site was amplified using a forward primer with an M13 sequence (marked in italics) at the 5′ end (5′-*TGTAAAACGACGGCCAGT*ATTGAAGGATGCCCTCTTTG-3′) and a reverse primer with a 5′-*GTGTCTT* (PIG-tailed) modification (5′-*GTGTCTT*CCCATCGTGAAGCTGTCTAA-3′) in combination with a M13 primer but labeled at its 5′ with the fluorescein amidites dye (FAM) primer (FAM-5′-GTAAAACGACGGCCAGT-3′). The remaining embryos were raised to adulthood and outcrossed with wild-type AB fish. Seven to eight embryos from each outcrossed pair were screened for mutations in the F1 generation. Two different alleles were selected: *appb*^26_2^ (−5 bp) and *appb*^26_4^ (−8 bp). These carry frameshift mutations and were raised and subsequently outcrossed towards the wild-type AB fish line until generation F4. Outcrossed adults were genotyped using a kompetitive allele-specific PCR (KASP) assay (LGC, Middlesex, UK). Offspring from heterozygous F4 inbreeds were used to generate homozygous wild-type and mutant lines. Embryos/larvae from these adult zebrafish were used to eliminate the influence of other potential genetic differences.

### Body length measurement

Microscopic images were acquired using a Nikon stereomicroscope (Mellville, NY, USA). For the one-cell stage (WT n = 10, *appb*^*26_2/26_2*^ n = 8), the size of the embryo was measured across the yolk. The total body length of 24 hpf (WT n = 20, *appb*^*26_2/26_2*^ n = 20), 48 hpf (WT n = 15, *appb*^*26_2/26_2*^ n = 19) and 3 dpf (WT n = 15, *appb*^*26_2/26_2*^ n = 19) embryos was measured from the apical part of the head to the caudal end of the tail fin, along the anterior-posterior axis. The 24 hpf was repeated once and other time points performed once.

### Staging of epiboly

Adult wild-type and *appb*^*26_2/26_2*^ fish were bred and eggs collected at matching time points were used to follow epiboly. Eggs were sorted out at one cell stage with wild-type eggs (n = 70) and *appb*^*26_2/26_2*^ mutant eggs (n = 72) placed in a separate dish with pre-warmed EM and allowed to develop at 28.5 °C. Both genotypes were inspected at time-point slightly preceding the reference time of zebrafish development and were kept on a heating plate when not in the incubator. The time of each stage was set when all eggs in a plate reached the set stage. The procedure was repeated three times. The differences in time for wild-type to reach dome stage compared to the reference time of 4.33 h was used to normalize the consecutive stages of both genotypes to correct for technical differences between repeats. A two-way ANOVA was used to examine the effect of genotype on epiboly progress. Post-hoc comparisons using Bonferroni’s multiple comparisons test was used to analyze the delay in mutants compare to wild-type at each specific stage using a confidence interval of 95%. Variation between repeats are shown as SD.

### *In situ* hybridization

Antisense digoxigenin-labeled RNA probes were generated from linearized DNA templates against *appb*^16,17^ and *aplp2* (MDR1734-202739496, Open Biosystems). *Appa* riboprobes were generated by amplifying fragments from cDNA clones, inserting them into the pCR2.1-TOPO vector (K450001, Invitrogen), linearizing the vector and transcribing with RNA polymerase using the following oligonucleotides: *appa*, Fwd 5′-AGGCGCATCGCGTTCTTCACAGAG-3′ and Rev 5′-CCTAACCCTCCCCGAACCCTCCC-3′. The aplp1 riboprobe was transcribed from PCR-amplified fragment using primers with T7 or T3 linkers (Fwd 5′-TAATACGACTCACTATAGGGTCGCGGTGTGGAATATGTCTGCT-3′ and Rev 5′-GCAATTAACCCTCACTAAAGGGTCTGCCTCTGCCCACTCCTTCA-3′). Whole mount RNA *in situ* hybridization (WISH) was performed as described earlier^18^ with modifications^17^. For each probe, wild-type and *appb* mutant embryos were anesthetized and then mixed in the same tube to be processed equally. In short, each sample was fixed in 4% PFA over night at 4 °C, permeabilized with proteinase K, blocked in hybridization mix and then incubated at 70 °C with the corresponding probe. After post-hybridization washes and colorimetric development of bound riboprobes the expression pattern was compared with wild-type and mutant larvae previously processed for WISH in separate tubes.

### Immunofluorescence and confocal microscopy

Embryos at sphere (4 hpf) and germ ring (5.7 hpf) stages were fixed overnight in 4% paraformaldehyde at 4 °C and then washed sequentially in phosphate-buffered saline (PBS), 0.5% Triton-X in PBS (PBT) and then dechorionated. Dechorionated embryos were incubated in block solution (5% normal goat serum, 1% bovine serum albumin [BSA], 1% DMSO and 0.5% TritonX-100 in PBS) for 2 hrs and incubated overnight (ON) at 4 °C in block solution containing 1:50 Phalloidin Alexa 568 (A12380, Molecular Probes) to stain actin, or antibody towards E-cadherin (610181, BD Bioscience), β-catenin (C2206, Sigma-Aldrich) or ZO-1 (33-9100, ThermoFisher Scientific). The next day, embryos were washed gently in PBT and incubated ON with secondary antibody with the desired fluorescent conjugate or were mounted in 1% agarose in glass bottom dishes (D29-10-1.5-N, Cellvis) in PBT medium and imaged using Zeiss LSM710 confocal microscope (Carl-Zeiss, Jena, Germany) using a 40x water immersion objective (Plan-Apochromat 40×/1.0) with 488 and 568 nm lasers.

For proliferation assay, wild-type and mutant embryos at 4 hpf were fixed in 4% PFA for 1.5 hrs at room temperature, washed with PBS and incubated with ice-cold 100% acetone at −20 °C for 20–30 min. Samples were transferred to a 24-well plate and washed five times with PBT on gentle agitation. Nonspecific binding was blocked with block solution (5% normal goat serum, 1% bovine serum albumin [BSA], 1% DMSO and 0.5% TritonX-100 in PBS) for at least 60 min and then incubated over night at 4 °C with primary antibody against phospho-histone H3 (pHH3, 06–570, Millipore) diluted 1:500 in block solution. Samples were washed carefully 5×10 min in PBST at RT and incubated with goat anti-rabbit- Alexa Fluor 488 (A-11008, Molecular Probes) over night at 4 °C or 2 hours at room temperature. Samples were carefully washed five times for 10 min in PBST. During the final wash, DAPI (D1306, Molecular Probes), 1:1000 and Alexa Fluor 568 Phalloidin (A12380, Molecular Probes) 1:300 were added to the PBST wash solution. Blastulas were mounted in 1% low melting agarose on glass bottom 35 mm Petri dish (81158, Ibidi). Stacks were acquired with an inverted Nikon A1 confocal system (Nikon Instruments, Melville, NY, USA) using a 20× objective. Quantification of proliferating EVL cells was performed manually by counting phalloidin- and pHH3-positive cells located at the outer cell layer of the blastula using ImageJ software (National Institutes of Health, USA).

### Quantification of cell shape /morphology

Confocal images of phalloidin-stained embryos were analyzed by ImageJ 1.52a software (National Institutes of Health, USA). Images were converted to binary and the threshold set manually to visualize cell borders. Noise was removed with Despecle plugin. Cell borders were outlined in binary images with the Watershed plugin. False borders were manually adjusted. Cell number, cell area and cell circularity were analyzed with the plugin Analyze particles. Junctions were manually counted. Image analysis were performed in a blinded fashion on decoded images.

### Antibody production

A polyclonal antibody (EER15) was produced by immunizing rabbits with a peptide corresponding to the N-terminal half of the zebrafish (Aβ) fragment (Ac-EERHNAGYDVRDKRC-CONH_2_) (Agrisera, Umeå, Sweden), since this sequence differs significantly between Appa and Appb. Antibodies from the second bleed were protein G-purified.

### Western blotting

Adult brains (10 months, n = 4) or deyolked embryos at 24 hpf (at least 60 per sample, n = 4) were used to analyze protein level. Protein level analysis was repeated at least twice. Brains and embryos were homogenized with a 23 G syringe and lysed in lysis buffer (10 mM Tris-HCl pH 8.0, 2% sodium deoxycholate, 2% SDS, 1 mM EDTA, 0.5 M NaCl, 15% glycerol) supplemented with protease inhibitor cocktail (04693132001, Roche). Samples were sonicated for 10 min and incubated on ice for 20 min. Supernatants were collected and protein concentration was measured using the BCA Protein Assay Kit (23225, ThermoFisher Scientific). Proteins were separated on a NuPAGE™ Novex™ Bis-Tris pre-cast gel (NP0321BOX, Invitrogen) and transferred onto 0.2 μm nitrocellulose membrane (GE Healthcare). The membrane was blocked with 5% milk and immunoblotted with anti-APP (Y188) antibody (ab32136, Abcam) at a dilution of 1:2000 (100% homology to zebrafish Appb and 93% to Appa) or the G-protein-purified, in house-generated polyclonal rabbit anti-Appb antibody, generated against the N-terminal half of the Aβ peptide. Immunoreactivity was visualized by anti-rabbit HRP-linked secondary antibody (7074 S, Cell Signaling) at 1:5000 dilution. The signal was developed using SuperSignal West Dura Extended Duration Substrate kit (Thermo-Fisher) and imaged using ChemiDoc Imaging Systems (Bio-Rad). Thereafter, blots were re-probed with 1:20000 GAPDH antibody (2D4A7) [HRP] (Novus Biologicals) or mouse anti-α-tubulin (T6199, Sigma-Aldrich) as a loading control. Western blot images were analyzed, produced and quantified using Image Lab™ Software (Bio-Rad). The intensity of the App band was normalized to the intensity of the loading control.

### Quantitative PCR

Between 10–50 of equally staged wild-type and *appb* mutant embryos were used per sample and total RNA was extracted using TRI Reagent^®^ (T9424, Sigma-Aldrich). Then, cDNA was synthesized using High Capacity cDNA kit (Applied Biosystems) with RNase inhibitor and converted in a single-cycle reaction on a 2720 Thermal Cycler (Applied Biosystems). Quantitative PCR was performed with inventoried TaqMan Gene Expression Assays with FAM reporter dye in TaqMan Universal PCR Master Mix with UNG. The assay was carried out on Micro-Amp 96-well optical microtiter plates on a 7900HT Fast QPCR System (Applied Biosystems). qPCR results were analyzed with the SDS 2.3 software (Applied Biosystems). Briefly, cDNA from each sample was normalized against average C_T_:s of the housekeeping gene *actb1* (Dr03432610_m1), then the relative quantity was determined using the ΔΔC_T_ method^19^ with the sample of wild-type sibling embryos (24 hpf) as the calibrator. Each sample was run in triplicates with at least 4 individual samples. Sample number used for each assay was *appa* (n = 15), *appb* (n = 8), *aplp1* (n=11) and *aplp2* (n = 13). TaqMan^®^ Gene Expression Assays (Applied Biosystems) were used for the following genes; Amyloid Beta (A4) Precursor Protein A (*appa*; Dr 031 443 64_m1), Amyloid Beta (A4) Precursor Protein B (*appb*; Dr 030 803 08_m1), Amyloid Beta Precursor Like Protein 1 (*aplp1*; AJCSWD2), Amyloid Beta Precursor Like Protein 2 (*aplp2*; Dr03437773_m1), Eukaryotic Translation Elongation Factor 1 Alpha 1, Like 1 (*eef1a1l1*; Dr 034 327 48_m1) and Actin, Beta 1 (*actb1*; Dr 034 326 10_m1).

### Ionomycin treatment

Wild-type and *appb* mutant embryos were treated with 5 μM ionomycin at the 32-cell stage (1.75hpf) and compared at the blastula stage (2.5hpf). Ionomycin (I0634, Sigma-Aldrich) stock was prepared in DMSO and dissolved in EM prior to exposure to achieve final 5 μM concentration. Similar concentration of DMSO without the compound was used as control.

### Cell dissociation and aggregation assay

Dissociation and aggregation of blastoderm cells were performed as described^20^ with some modifications. Embryos were injected at the one- to four-cell stage with 10 μg/μl of 10’000 Mwt dextran-Alexa Flour 488 (D22910, Molecular Probes**)** or tetramethylrhodamine-dextran (D1824, Molecular Probes**)** diluted in 0.2 M KCl and filtered through a 0.22 μm filter. At 4 hours post fertilization, embryos were manually dechorionated on an agarose plate and transferred to a deyolking buffer (55 mM NaCl, 1.8 mM KCl, 1.25 mM NaHCO_3_). Embryos were dissociated by pipetting up and down using a glass Pasteur pipette. Blastoderm cells were harvested by centrifugation at 400xg for 3 min at RT. Cells were washed twice with sterile DPBS (14190086, Invitrogen), resuspended in L15 medium (11415049, ThermoFisher), containing 2 mM L-glutamine (G7513 Sigma-Aldrich, Munich, Germany), 100 U/ml Penicillin, 100 μg/mL Streptomycin (P4333, Sigma-Aldrich), 10% FBS and 1.25 mM CaCl_2_. The cell suspension was transferred through a 40 μm cell strainer (734-0002, Corning) and transferred to a glass bottom 35 mm Petri dish (81218, LRI Instrument AB,) to a final concentration of 50 embryos/35 mm dish. Petri dishes were coated with 10 μg/ml fibronectin (11051407001, Sigma-Aldrich) at 37 °C for at least 2 h and washed with DPBS to remove excessive fibronectin. Cell cultures were incubated at 31 °C in an incubator or heating chamber on the confocal microscope while cluster formation was analyzed. Confocal planes were taken at 1, 6 and 16 hours post dissociation (hpd) with a 10× objective (Plan Apochromat 10x/0.45NA) on a Nikon A1 Confocal Microscope System (Nikon Instruments, Melville, NY, USA). Three separate experiments (*n* = 3) performed on larvae from different parents were used for image analysis.

#### Analysis of aggregation and segregation

Collected images were treated as a matrix of dimensions x,y,z, where x is the number of row pixels, y is the number of column pixels and z is 2 - the number of color channels (red and green). To automatically segment the aggregates in the field of view, maximum intensity z-projection images were processed in FIJI software as such: contrasts were enhanced via the Normalize Local Contrast method. Smoothing was done via a Gaussian Blur of sigma equals 1. Image thresholding was done via Li’s Minimum Cross Entropy method. Each aggregate was then identified as a proper region of interest (ROI) and objects smaller than 1000 pixels were removed from the segmented image (software tools for segmentation and quantification are available at: https://github.com/CamachoDejay/). To characterize the segregation of the aggregates, we implemented a method previously described^21^. In brief, P (the electrical dipole moment) and S (the moment of inertia and ratio of scattering amplitudes) parameters were calculated. For this, each image color channel, red and green, was loaded as a separate matrix, where the matrix value indicated intensity and the row and column index represented the pixel position (intensity matrix l[r,c], where ‘r’ and ‘c’ are row and column indices). A full description of S and P calculations can be found in supplementary materials (Supplementary data 5). Data from three technical repeats (*N* = 3) of clusters from a total of wt^(G)^/wt^(R)^ (*n* = 742) or wt^(G)^/mut^(R)^ (*n* = 1161) were plotted in GraphPad Prism® 7.

### Behavioral analysis

Adult heterozygous *appb* carriers (F3) were inbred to facilitate the study of locomotion of wild-type (*n* = 30) and mutant (*n* = 23) siblings. Larvae were incubated at 28.5 °C on a 14:10 h light to dark cycle. At 6 dpf, larvae of mixed genotypes were placed in 1 mL EM in a 48-well plate and acclimatized overnight in the behavior room at 26 °C and subsequently 20 min in the ZebraBox before initiation of tracking. Locomotor activity was recorded using the ZebraBox tracking system (ViewPoint, Lyon, France), equipped with infrared digital camera. Recording was carried out for 60 min in continuous light. Larvae were genotyped with a KASP assay (LGC, UK) specific for the mutation. The experiment was repeated twice. Swimming behavior was quantified using ZebraLab^TM^ software (ViewPoint, Lyon, France), values were calculated in Microsoft Excel and graphs were plotted in GraphPad Prism® 7.

### Statistical analysis

Statistical analysis was performed using GraphPad Prism^®^ 7 software. Data were presented as means with standard deviation (±SD) or standard errors of the mean (±SEM). For analysis of body length, western blot, locomotor activity, qPCR data, cell shape, proliferation and analysis of scattering and dipole moment were performed with unpaired Student’s *t*-tests. Analysis of epiboly progress was made with two-way ANOVA. Statistical significance was set at *p* < 0.05 (*), 0.01 (**), 0.005 (***) and 0.0001 (****).

## Results

### appb mutants show morphological defects at early stages

**Figure 1:**
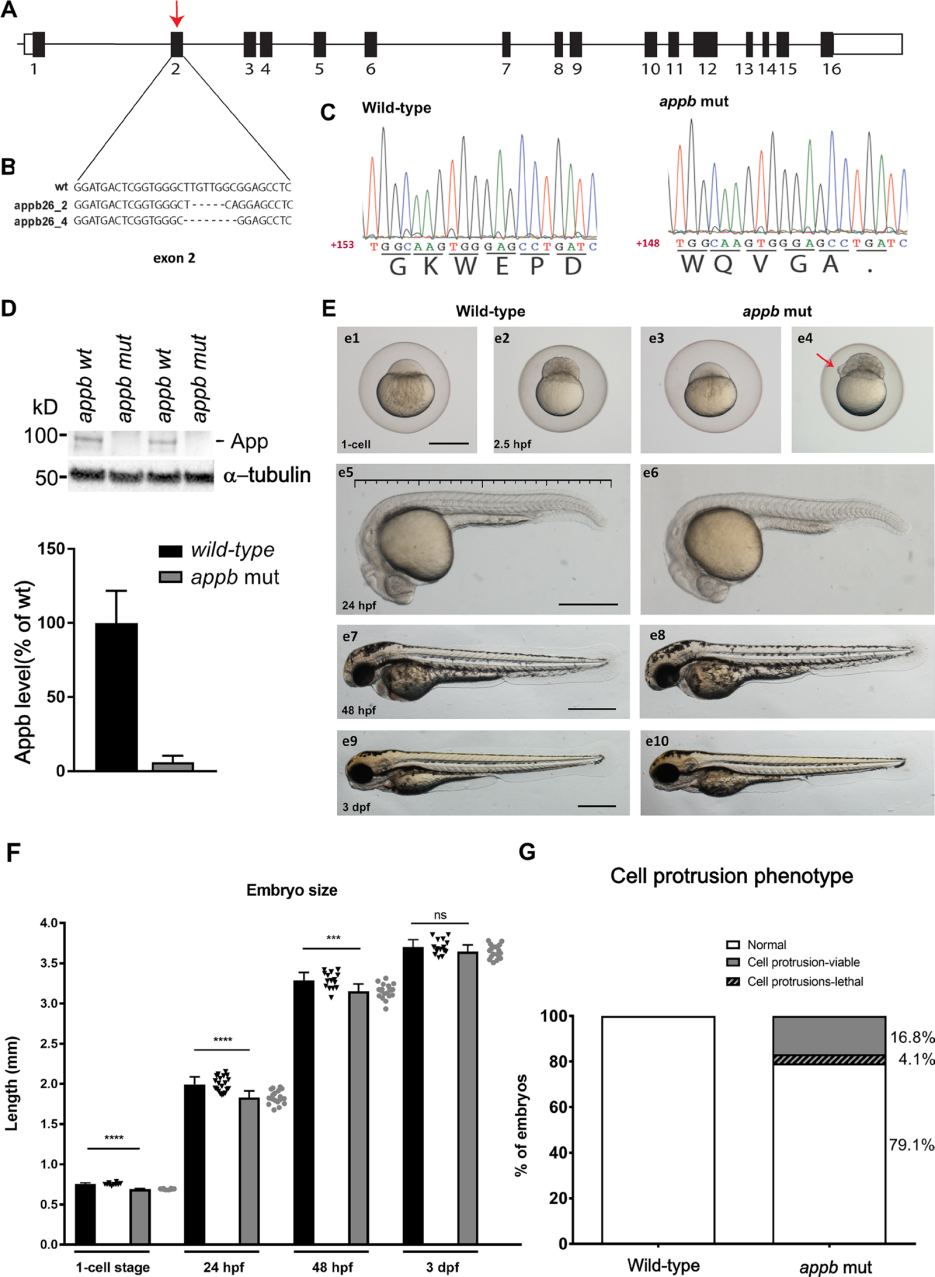
Characterization of the *appb* mutation. (**A**) Schematic representation of the coding (box) and non-coding regions (line) of the *appb* gene, indicating the mutation point in exon 2 (red arrow). (**B**) Nucleotide sequence surrounding the deletions (dashed line indicates missing nucleotides). (**C**) Sanger sequencing chromatogram of the 3´end of exon 2 in wild-type and *appb* mutants shows a premature stop (dot) in the translated protein sequence in mutants. (**D**) Western blot analysis of Appb expression in adult brain from wild-type (*n* = 4) and *appb* mutants (n = 4) using the in house-generated Appb-specific antibody (EER15) and α-tubulin as loading control. Quantification shown as percentage of wild-type. (**E**) Morphology of *appb* mutants and wild-type zebrafish embryos at one-cell stage to 3 dpf larvae (e1, e3, e5, e7, e9 indicates wild-type and e2, e4, e6, e8, e10 are *appb* mutants). Scale in e5 shows measurement of larvae. (**F**) Embryo size measurement at the 1-cell stage (wt, n = 10; *appb*^*26_2/26_2*^, n = 8), 24 hpf (wt, n = 20; *appb*^*26_2/26_2*^, n = 20), 48 hpf (wt, n = 10; *appb*^*26_2/26_2*^, n = 8), the size of the embryo was measured across the yolk. The total body length of 24 hpf (wt, n = 20; *appb*^*26_2/26_2*^, n = 20), 48 hpf (wt, n = 15; *appb*^*26_2/26_2*^, n = 19) and 3 dpf (wt, n = 15; *appb*^*26_2/26_2*^, n = 19) and 3 dpf (wt, n = 15; *appb*^*26_2/26_2*^, n = 19). Error bars are in SD and p–values calculated by t-test. Data points are given to the right of each bar with triangles (wt) or filled circles (*appb* mut). (**G**) Quantification of cell protrusion phenotype shown as percentage of all embryos. Wild-type (n = 418) and *appb* mutant (n = 465). Scale bar= 500 μm. *p < 0.05, **p < 0.01 and ***p < 0.001.

We established two *appb* mutant lines with the CRISPR/Cas9 method. The gRNA construct targeting the 5′-end of exon 2 (depicted with an arrow, Fig. 1A) generated a deletion of five nucleotides (dashed line) and insertion of two nucleotides (lower case letters) referred to as *appb*^*26_2*^ and a deletion of eight nucleotides as in *appb*^*26_4*^ (Fig. 1B). Sanger sequencing was used to confirm the mutations and showed disrupted reading frame and a premature stop codon at the 3′-end of exon 2 (amino acid 55, indicated by a black dot), as represented by the chromatograms (Fig. 1C). Both alleles showed the same phenotype and we therefore used allele *appb*^*26_2*^, hereafter referred to as the *appb* mutant.

We then examined the effect of the *appb* mutation on protein expression by western blot. We produced a polyclonal antibody towards the N-terminal part of the Aβ peptide, since we could not find any good commercially available antibodies that were specific to the zebrafish Appb protein. The N-terminal part of the Aβ region shows low homology between Appb and the other members of the App family and was therefore considered as a good candidate peptide to use for immunization. While protein with the expected size was found in wild-type brains, no bands were detected in *appb* mutant brains (Fig. 1D, Supplementary data 1D). Quantification using α-tubulin as a loading control to adjust for differences in protein loading showed a 95% reduction in Appb protein expression of mutants.

**Figure 2:**
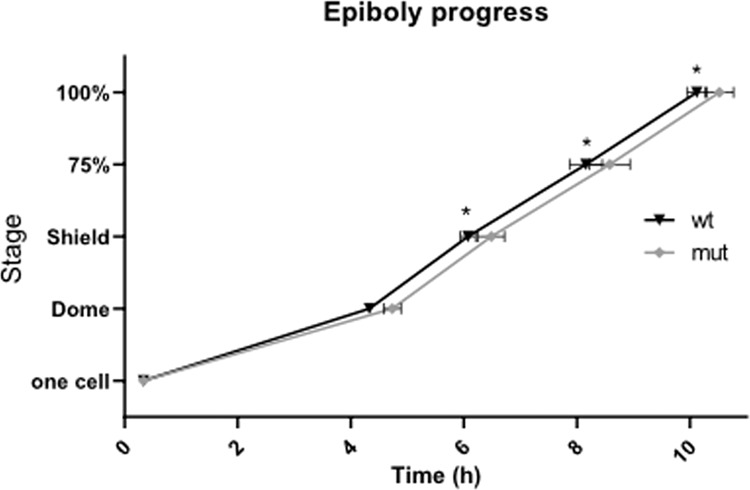
Delayed epiboly progression in *appb* mutants. The time (h) for wild-type (*n* = 70) and *appb* mutant (*n* = 72) zebrafish to reach 100% epiboly. Staging was initiated when embryos reached one-cell. Time for wild-type embryos to reach dome stage was set to 4.33 and mutants were normalized accordingly to allow for repeat comparison. The effect of genotype on epiboly progression was examined by two-way ANOVA(*p < 0.05) and as reported as mean ± SD. Post-hoc comparisons using Bonferroni’s multiple comparisons test indicated a significant difference in the delay in mutants compare to wild-type in all stages after dome stage with a p < 0.001.

Next, we performed gross morphological analysis of *appb* mutants from the 1-cell stage to 3 dpf larvae. The *appb* mutants, generated from homozygous parents, were significantly smaller at the 1-cell stage compared with wild-type embryos and had shorter body length until 48 hpf (Fig. 1E: e1-8; and Fig. 1F). In addition, we observed cells protruding through or perching on the EVL in ~20% of mutant blastulas (Fig. 1E: e4, red arrow and Fig. 1G), out of which some resulted in abnormal blastula formation (Supplementary data 1E). This phenotype was detected around 2.25 hpf when the EVL just has formed. While 4.1% of these embryos later died (Fig. 1G, Supplementary data 1G), the remaining embryos developed further and survived, although with a delay in epiboly of between 20 and 30 min *p* < 0.05 compared with wild-type fish (Fig. 2, Supplementary data 2). Intriguingly, no other gross morphological changes were observed at later stages compared with wild-type fish (Fig. 1E: e9-e10 and Fig. 1F).

### Defects in EVL integrity

**Figure 3:**
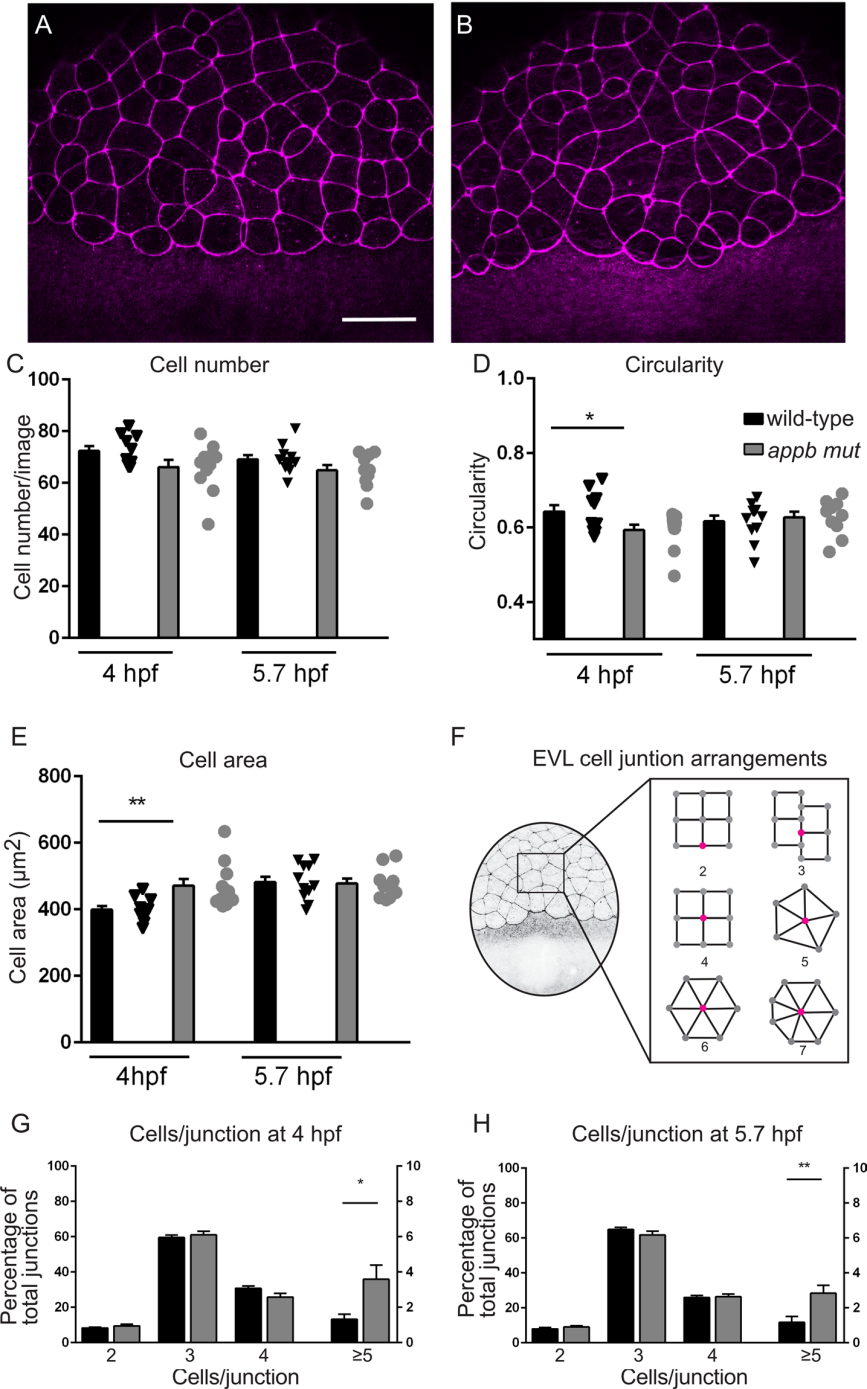
Changes in cell shape morphology in *appb* mutants. (**A**) Wild-type and (**B**) mutant embryos stained with phalloidin at 4 hpf. (**C–E**) Quantification of cell number and morphology. (**F**) Schematic of embryo at blastula stage and junction points connecting to number of cells. (**G,H**) Quantification of cells per junction points at sphere and germ ring stage. (Sphere: *n* = 12 wt, 12 *appb* mut and germ ring: *n* = 11 wt, 10 *appb* mut). Student’s *t*-test was used to analyze data. *p < 0.05 and **p < 0.01.

**Figure 4:**
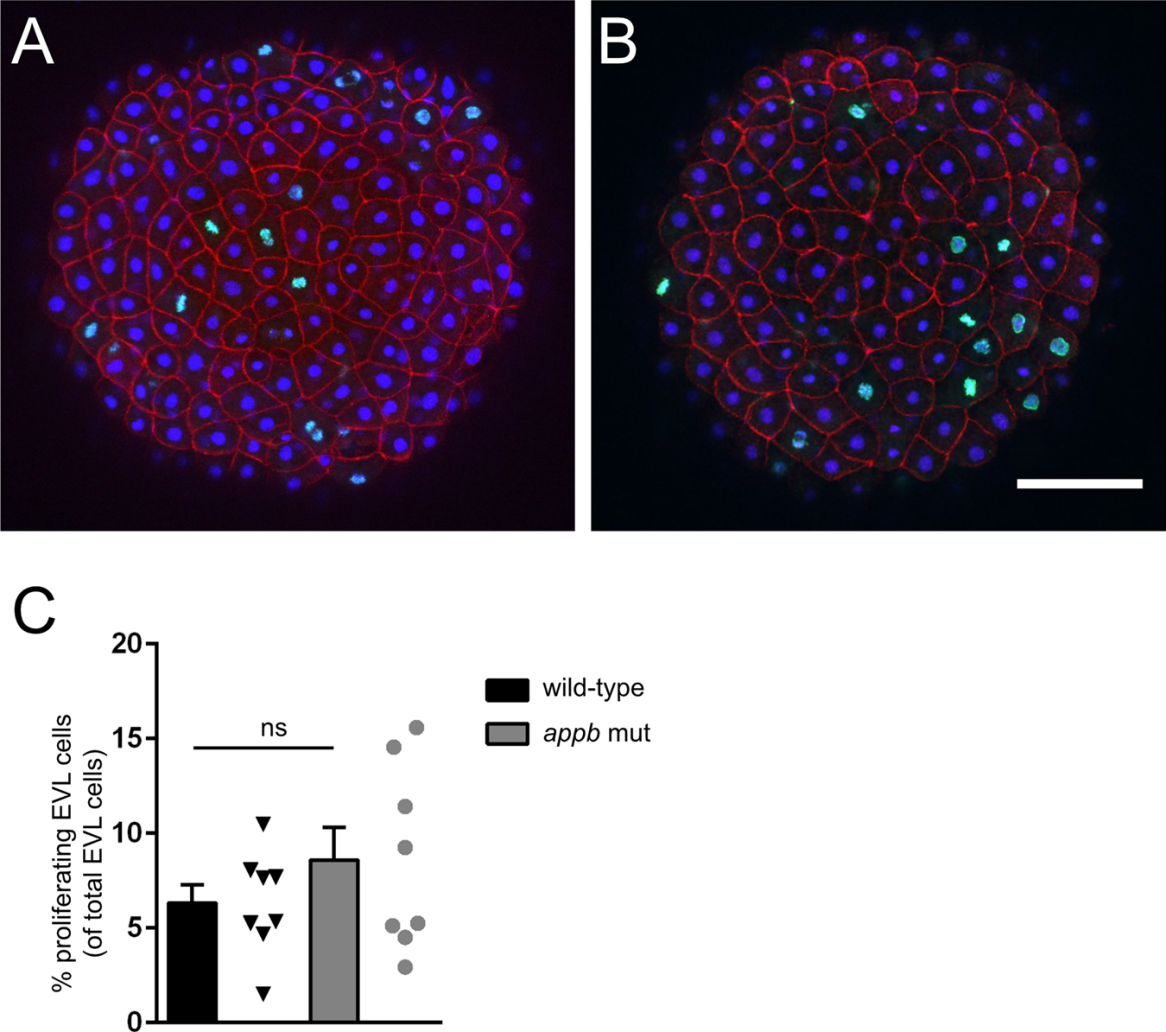
Proliferation of EVL in wild-type and *appb* mutants at 4 hpf. Representative confocal images of the EVL in wild-type (**A**) and *appb* mutant (**B**) embryos immunostained for the mitotic marker phospho-Histone H3 (pHH3; green) and actin binding phalloidin (red) at 4 hpf. All nuclei were labeled with DAPI (blue). Number of pHH3-positive EVL nuclei shown in percentage of all EVL cells (**C**). Student’s *t*-test was performed on wild-type (*n* = 8) and *appb* mutant (*n* = 8). Scale bar, 100 μm.

Similar phenotypes, including protrusions and delayed epiboly, have previously been described in other mutants with EVL defects^22–25^. We therefore continued with analyzing the cellular arrangement of the EVL and performed phalloidin-staining of actin organization at the sphere (4 hpf) and germ ring stages (5.7 hpf), as these stages are easy to distinguish and thus allow for accurate staging (Fig. 3A,B). Interestingly, EVL cells in *appb* mutants had a larger cell surface area and were less circular compared with EVL cells of wild-type blastulas at 4 hpf (Fig. 3D,E). At the sphere stage, the cell cycle of the EVL slows down significantly^26^ and the EVL is suggested to spread mainly by cell flattening^27^. However, if the EVL consists of fewer cells, the remaining cells would need to stretch more to cover the same area. We therefore analyzed whether the increase in EVL cell surface in mutants was due to decreased proliferation and thus changes in EVL cell number. However, we could not find any difference in the total number of EVL cells (Fig. 3C) nor in the number of EVL cells going through mitosis by staining wild-type (Fig. 4A,C) and mutant (Fig. 4B,C) blastulas for phospho-histone H3 (pHH3), DAPI (nuclei) and phalloidin (F-actin) at 4 hpf (Supplementary data 4).

The process of epiboly, starting shortly after sphere stage, is an orchestrated event depending on tissue expansion and radial intercalation of the blastoderm^28^. These processes are proceeding under surface pressure, coordinated by the cells of the EVL through cell rearrangements and cell divisions^28^. Thus, to understand the consequence of changed EVL morphology on cell arrangement, we quantified the number of cells per junction point, where a junction point was defined as the intersection between several cell membranes. (Fig. 3F, diagrammatic representation). In the wild-type blastula, at 4 and 5.7 hpf, we observed, most frequently, 3 or 4 cells sharing one junction point (Fig. 3G,H). A smaller percentage of all junctions were shared by only two cells. These were mainly present at the EVL margin close to the yolk syncytial layer. These EVL junctional formations were similar between *appb* mutant and wild-type (Fig. 3G,H). In addition, we found rosette-like junction arrangements with 5 cells in wild-types. However, mutants had significantly more rosette-like formations with up to 7 cells connecting to one junction point (Fig. 3G,H). Together, these results show that mutants have abnormal EVL morphology and arrangement. Such changes might be affected by defects in cell adhesion^24^, and since APP act as an adhesion molecules at synapses^29^, we hypothesized that cell-shedding together with changes in cell shape might reflect failing cell-cell adhesion.

### Increased segregation of Appb mutant cells in an *in vitro* aggregation assay

**Figure 5:**
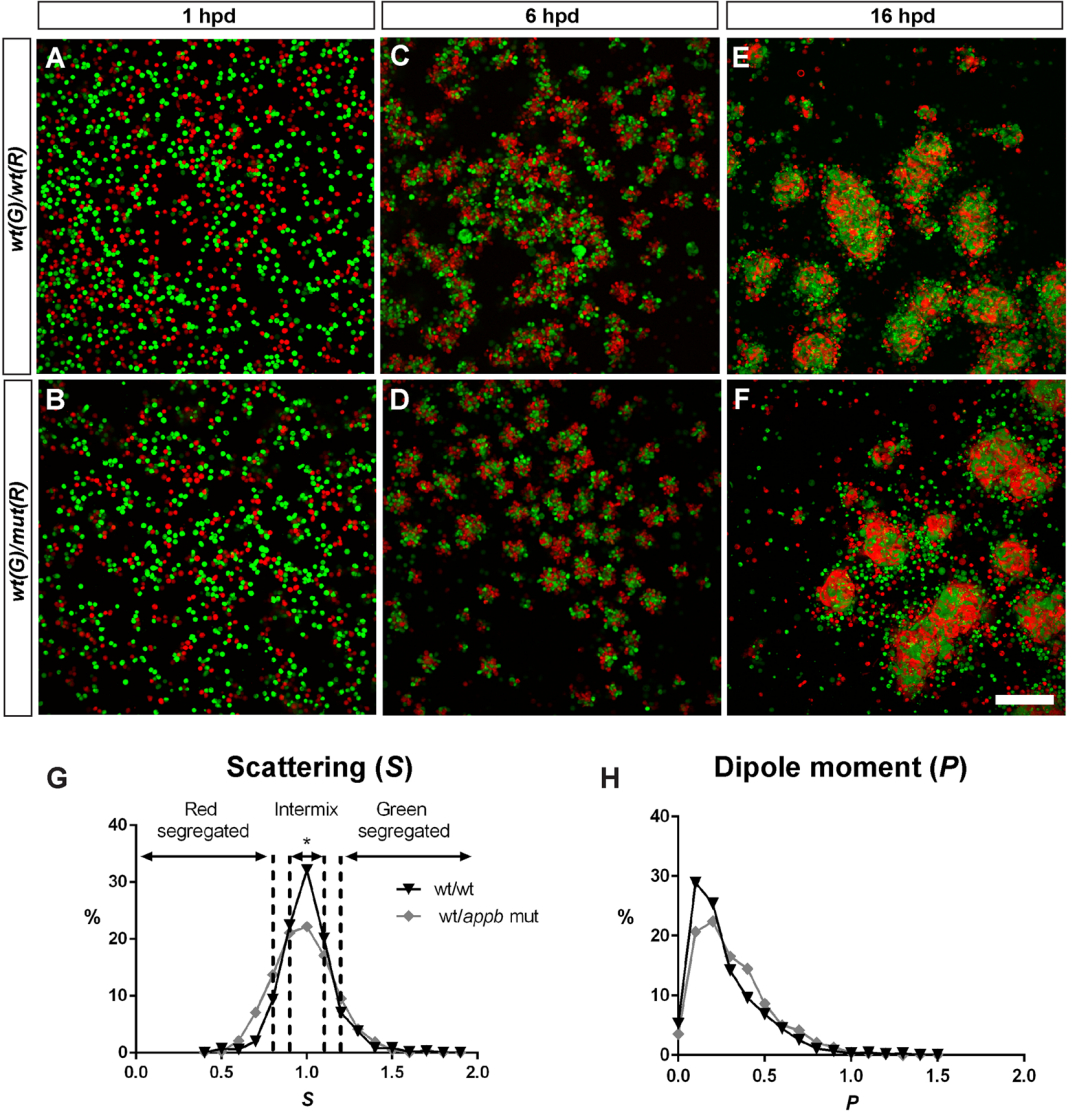
Appb regulates blastoderm cell adhesiveness. Embryos of wild-type or *appb* mutant background were injected with red (R) or green (G) fluorescent dextran at the one-cell stage, dissociated and mixed 1:1 at sphere stage and co-cultured in fibronectin coated plates (**A,B**). Cluster formation started around 6 hours post dissociation (hpd) (**C,D**) and was allowed to proceed for 16 hpd before image analysis (**E,F**). Scale bar = 200 μm. Frequency distributions of scattering ‘S’ (**G**) and dipole moment ‘P’ (**H**) of red and green cells in clusters of wt/wt (black line) and *wt*/*appb* mut (grey line). Dashed lines mark interval for scattering. *p < 0.05.

Differences in cell-cell adhesion can be evaluated by an aggregation test where dissociated cells are allowed to re-aggregate *in vitro*. In such experimental setup, cells with different adhesiveness will segregate from each other, while cells with equal adhesiveness will mix randomly^20,21,30–32^. To address if the adhesive property of *appb* mutant cells are different from wild-type cells, we therefore carried out a cell dissociation and re-aggregation test. Wild-type and mutant embryos were labeled by injections of red (R) or green (G) fluorescent dextran at the one cell stage, dissociated and plated at a 1:1 ratio in combinations of wt^(G)^/wt^(R)^ and wt^(G)^/mut^(R)^ (Fig. 5A,B). Although, aggregates started to form at 6 hours post dissociation (hpd) (Fig. 5C,D), the segregation process was allowed to proceed until 16 hpd (Fig. 5E,F). At this stage, both wild-type and *appb* mutant cells adhered to form well-defined clusters. Clusters containing wt^(G)^/wt^(R)^ cells (*n* = 742) or wt^(G)^/mut^(R)^ cells (*n* = 1161) were intermixed and did not show the clear segregation patterns as previously reported between cells with large differences in adhesion^21–23,33^. On the other hand, the mild phenotype of *appb* mutants predicted a less dramatic change in the segregation process. Thus, to analyze more subtle changes in cell distribution, we took advantage of the image analysis method developed by Schötz and colleagues^21^, which uses two parameters to quantitatively define scattering (S) and dipole moment (P) of cells within clusters. S is defined as S = S_red_/S_green_ where an S close to 1 represent intermixed cells and close to 0 or 2 when one cell group is segregating from the other. On the other hand, P describes separation of two populations and is close to 1 if cells in a cluster are completely separated into a red and green compartment but close to 0 when cells are fully mixed or when one cell type surround the other.

When applying these parameters to our data, we found a significantly lower level of intermixed (0.9 > S < 1.1) clusters in wt^(G)^/mut^(R)^ (43%) compared to wt^(G)^/wt^(R)^ clusters (56%, p < 0.05), as shown in the frequency diagram (Fig. 5G). In addition, the shape of the S-curve has a tendency of being broader, suggesting a higher percentage of low S-values in clusters with mutant cells. This, in combination with the tendency of P being shifted towards 1 in wt^(G)^/mut^(R)^ clusters, shows increased segregation in clusters with wt and *appb* mutant cells and that these even may separate from each other, supporting a role of Appb in cell adhesion.

### appb mutants are sensitive to ionomycin treatment

**Figure 6:**
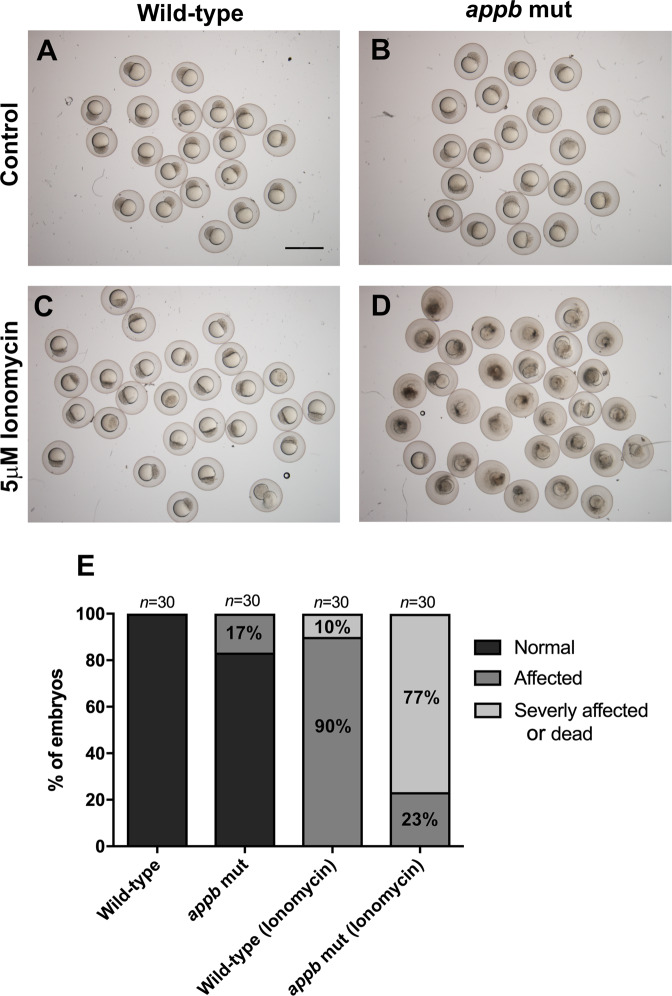
Increased sensitivity to ionomycin treatment in *appb* mutants. (**A,B**) Wild-type and *appb* mutant embryos treated with DMSO control. **(C,D**) 5 μM ionomycin treatment of wild-type and *appb* mutants embryos. (**E**) Quantification of ionomycin sensitivity. Scale bar, 1500 μm.

To investigate if the observed perturbations were a consequence of defective cell-cell interactions *in vivo*, we tested if mutant embryos were more sensitive to additional deterioration of cell adhesion. Ionomycin is a Ca^2+^ influx stimulator that promotes shedding of the adherens junction proteins cadherins^34,35^, which leads to cell-cell detachment. We hypothesized that although not all mutant embryos displayed cell protrusions and shedding, they might still be more sensitive to ionomycin exposure. We treated wild-type and *appb* mutants with 5 μM ionomycin at the 32-cell stage (1.75 hpf), observed them at the blastula stage (2.5 hpf) and quantified the phenotypes based on severity (Fig. 6). Wild-type embryos treated with control DMSO showed no phenotype, while ~17% of mutant embryos showed cell adhesion phenotypes similar to incubation in EM (Fig. 6A,B,E). Interestingly, we found that around 77% of mutant embryos were severely affected/dead by the ionomycin treatment (Fig. 6D,E) compared with 10% of ionomycin-treated wild-type embryos (Fig. 6C,E). These data indicate that mutant embryos are more sensitive to additional weakening of cell-cell adhesion compared with wild-type embryos, supporting the hypothesis that Appb affects cell-cell interactions either directly or through other adhesion molecules.

### No change in expression of tight- or adhesion junction markers ZO-1, E-cadherin and β-catenin

**Figure 7:**
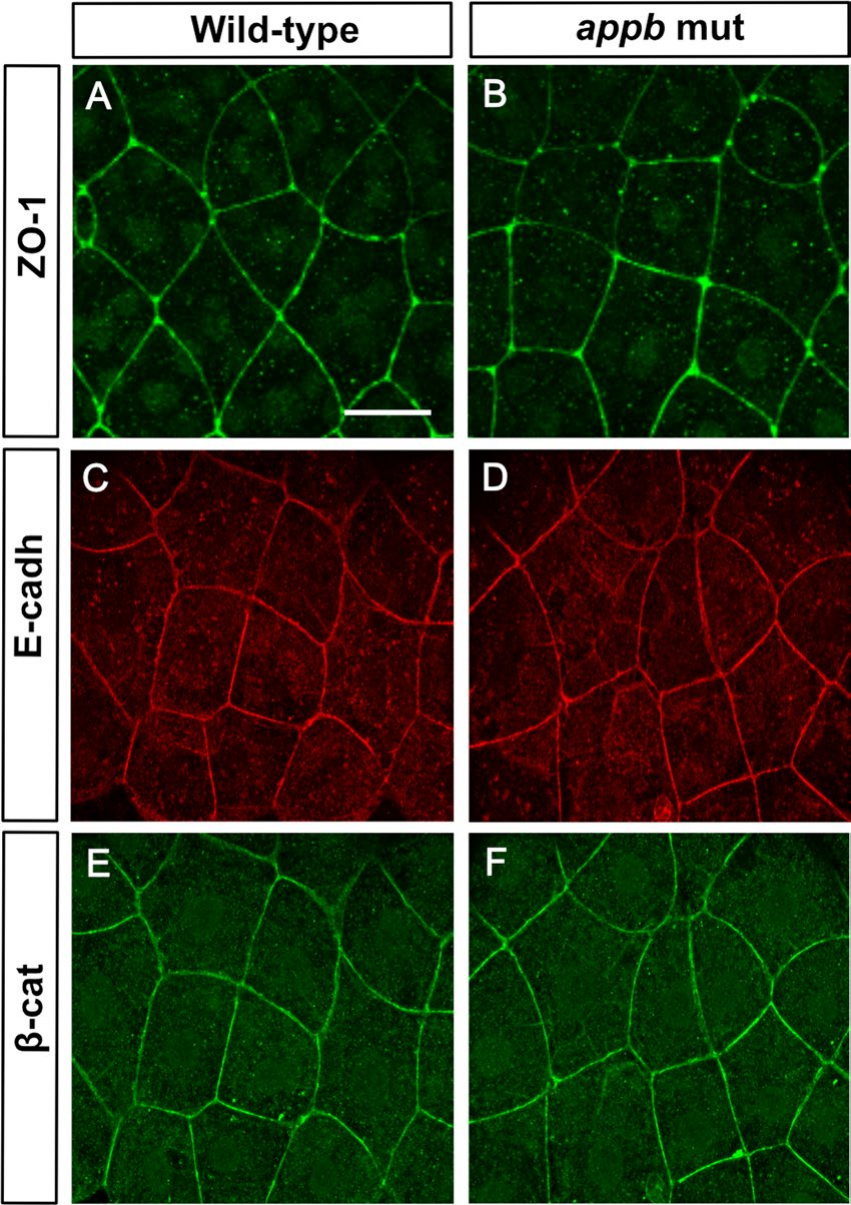
Normal expression of ZO-1, E-cadherin and β-catenin at 5.7hpf in *appb* mutants. Wild-type and mutant embryos at 5.7 hpf stained with ZO-1 (**A,B**), E-cadherin (**C,D**) and β-catenin (**E,F**). Wild-type (*n* = 11) and *appb* mutant (*n* = 12). Scale bar = 25 μm.

The above results led us to examine the arrangement of the cell-cell junctions. We stained embryos at 5.7 hpf for ZO-1, a protein expressed at tight junctions, and E-cadherin, a protein present in adhesion junctions. However, we were not able to detect any differences in neither the level or distribution pattern of either protein between wild-type (Fig. 7A,C) and *appb* mutants (Fig. 7B,D). As E-cadherin establishes connections with the cytoskeleton through binding β-catenin^36,37^, we also analyzed the expression of β-catenin in embryos at 5.7 hpf and found no obvious change between wild-type and mutant (Fig. 7E,F). Together, these results suggest that the general structures of both tight and adherens junctions are intact and that E-cadherin, ZO-1 and β-catenin do not seem to be affected by the mutation in *appb*.

### Upregulated transcription of other App-family genes

The apparently normal phenotype of *appb* mutants at later stages indicate activation of compensatory processes. To investigate if the expression of other *app* family members was upregulated, we performed whole mount *in situ* hybridization and qPCR on wild-type and *appb* mutants for mRNA expression of *appa, appb, aplp1* and *aplp2*.

**Figure 8:**
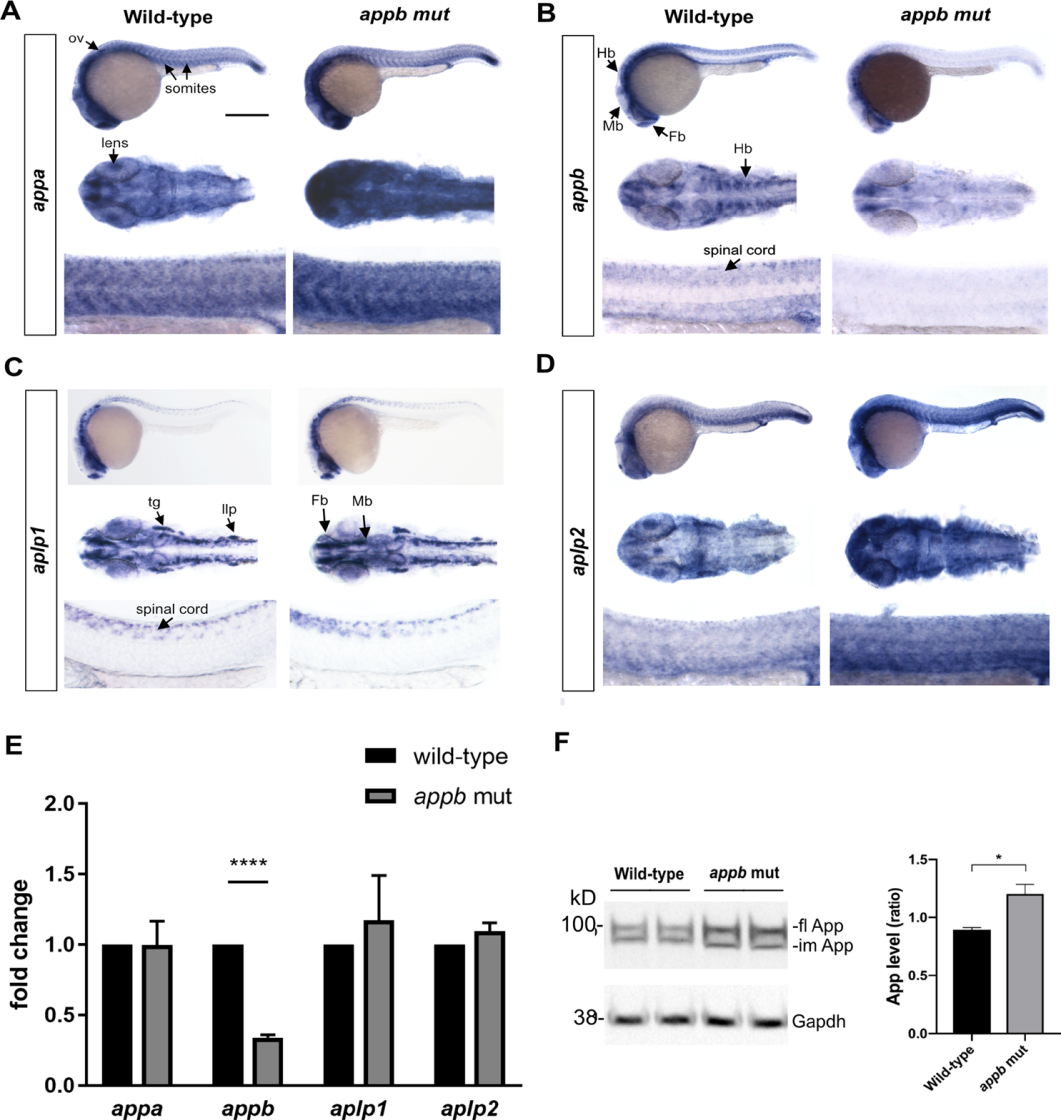
Expression of app family in *appb* mutants. Whole mount *in situ* hybridization of *appa* (**A**), *appb* (**B**), *aplp1* (**C**) and *aplp2* (**D**) at 24 hpf. Upper panel: whole embryos; middle panel: head (dorsal view) and lower panel: trunk in each set of genes (anterior to left). Scale bar =500 μm (upper panel), 100 μm (middle panel) and 50 μm (lower panel). (**E**) Whole embryo qPCR analysis of *appa*, *appb*, *aplp1* and *aplp2* at 24 hpf. Values are reported as mean ±SEM. ****p < 0.001. (**F**) Western blot analysis and quantification of zebrafish App expression in appb mutants (n = 4) at 24 hpf using the Y188 antibody shown as ratio of wild-type (*n* = 4). Mean is reported as ± SD. Fl, full length; im, immature. *p < 0.05, **p < 0.01 and ****p < 0.001. ov, otic vesicle; Fb, forebrain; Mb, midbrain; Hb, hindbrain; llp, lateral line primordium; tg, trigeminal ganglia.

At 24 hpf, *appa* expression is found in tissues of mesodermal origin, lens, otic vesicles and somites^38^. We observed a general increase in the expression of *appa* mRNA in *appb* mutants compared with wild-type embryos (Fig. 8A, upper panel). When we analyzed the expression, we found that *appa* mRNA was highly increased in both head (Fig. 8A, middle panel) and somites of *appb* mutants (Fig. 8A, lower panel).

The expression of *appb* transcript is reported as localized to axial structures and is strongly expressed in neural tissues including telencephalon, mesencephalon hindbrain, hindbrain rhombomeres, trigeminal ganglia and spinal cord^16,17,38^. Here we found that the expression of *appb* mRNA was drastically reduced in *appb* mutants compared with their wild-type siblings (Fig. 8B, upper, middle and lower panel). In particular, nearly no expression was observed in the hindbrain and spinal cord region (Fig. 8B, middle and lower panel), confirming that the mutation results in increased degradation of *appb* mRNA.

The *aplp1* and *aplp2* genes are not well studied in zebrafish. Whole mount *in situ* hybridization revealed that *aplp1* was expressed in sensory ganglia including trigeminal ganglia and lateral line primordium of the developing brain and spinal cord (Fig. 8C). Strong expression was also observed in the telencephalon and mesencephalon. No visible change in *aplp1* mRNA expression was detected in *appb* mutants (Fig. 8C, upper, middle and lower panel). In contrast, *aplp2*, with an mRNA expression pattern similar to *appa*, was observed to have a general increase in head and trunk of *appb* mutants as compared to the wild-type embryos (Fig. 8D, upper, middle and lower panel).

Quantification of *appb* and *aplp1* expression in whole embryos by qPCR correlated well with the above results (Fig. 8E). However, the apparent upregulation of *appa* and *aplp2* in spatially specific regions observed with *in situ* hybridization could not be validated when addressed on whole embryo extracts (Fig. 8E).

These data led us to address App protein expression in mutants using the Y188 antibody that in mice only bind to APP and not APLP2^39^. Western blot on whole embryos at 24 hpf showed increased protein levels in *appb* mutants compared with wild-type embryos (Fig. 8F). This increase is likely representing Appa since the C-terminus (last 20 amino acids, including the peptide used in antibody production, Supplementary data 8) of Aplp2 in zebrafish is 100% conserved between and mouse. Thus, if Y188 does not bind APLP2 in mice, we would expect the same in zebrafish. However, the specificity of Y188 in zebrafish needs further evaluation on other zebrafish App family mutants to be fully supported.

In summary, our results suggest that the loss of Appb protein in *appb* mutants is accompanied by increased expression of Appa and, possibly also Aplp2. The increase in expression likely compensates for the deficits seen in mutants at early developmental stages.

### Normal locomotion at larval stage

**Figure 9:**
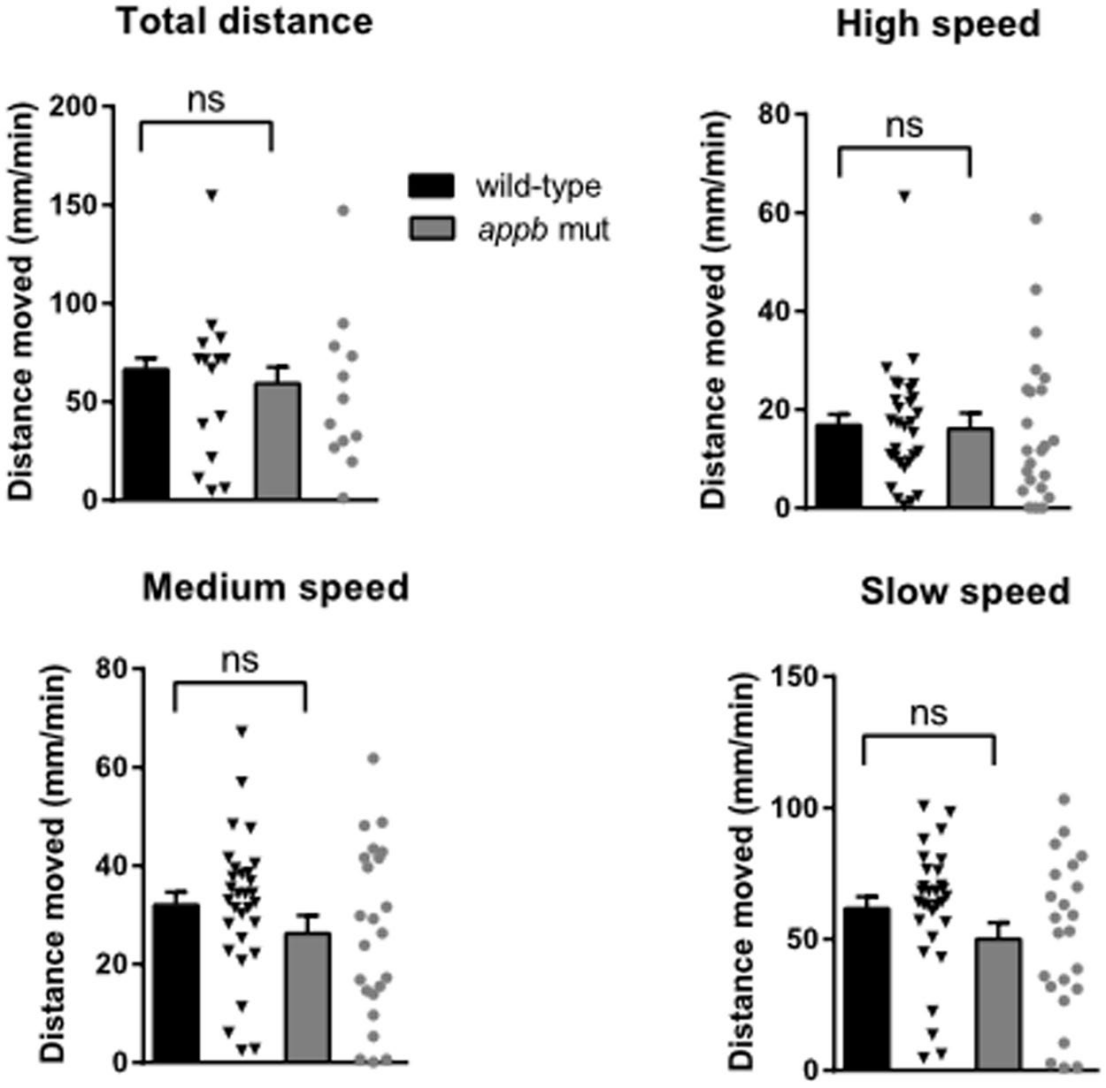
Normal locomotor activity in *appb* mutants at 6 dpf. Swimming activity over 60 minutes displayed as total distance traveled per minute (**A**), or as distance traveled at high (**B**, <6.1 mm/s), medium (**C**, 2.1–6.1 mm/s) and low (**D**, >2.1 mm/s) velocity. Wild-type (n = 30) and *appb* mutant (n = 23). Data were analyzed using Student’s *t*-test and reported as mean ± SEM.

To investigate whether the absence of Appb protein leads to behavioral defects, we monitored locomotor activity in the zebrafish larvae. To that end, we bred F3 heterozygous parents to generate F4 offspring. Comparison of the locomotor activity of the wild-type and *appb* mutants at 6 dpf revealed no significant difference in the distance traveled by *appb* mutants and wild-type larvae recorded over a 60-min time frame (Fig. 9A). In addition, the assessment of distance traveled at different velocity [high (>6.1 mm/sec), medium (2.1–6.1 mm/sec) and slow (<2.1 mm/sec)] did not show significant difference between these two groups (Fig. 9B–D).

## Discussion

In the present study, we describe zebrafish with a homozygous mutation that introduces a premature stop in exon 2 of the *APP* homologue, *appb*. Similar to *App* mutant mice^12,13^, zebrafish lacking *appb* were initially smaller but morphologically normal, healthy and fertile as adults. Due to the external development of zebrafish embryos, we were able to identify a phenotype previously not described, which indicates a role of *appb* in cell adhesion during early development. The physiological function of APP family members has been hard to discern, likely due to the apparent redundancy between the APP and APLP2. As zebrafish have two *APP* orthologues, *appa* and *appb*, in addition to the *aplp* genes, we expected *appb* mutants to have a similar or even milder phenotype than that described in mice. Indeed, this seems true after gastrulation. However, during early development, disrupted blastula integrity, together with general changes in EVL morphology and delayed gastrulation, clearly indicates the importance of Appb in these processes.

During the initial phase of gastrulation, the EVL is suggested to control thinning and extension of the underlying blastoderm cells, a process known as doming, through adjustments of surface tension^28^. This process, as well as later stages of gastrulation, depends on tightly controlled cell-cell adhesion both within the EVL and in contact with the underlying blastomeres. Defects in EVL integrity are typically seen in zebrafish with genetic mutations affecting adherens junction, tight junction and cytoskeletal proteins^24,25,40–42^. Here we suggest that Appb is important for the cellular rearrangements during gastrulation. Although only a small fraction of *appb* mutant embryos displayed visible cell protrusions, their general sensitivity to additional destabilization of cell adhesion, suggest that cell-cell interactions are weaker in mutants. Accumulating evidence shows that E-cadherin-mediated adhesion is crucial for proper gastrulation^23,43–46^. Interestingly, our *appb* mutants shared several phenotypes with E-cadherin hypomorphs (*hab*^*rk3*^), including uneven blastula surface, detaching cells and delayed epiboly^46^. In addition, Appb knockdown experiments report convergence/extension and sensory neuron defects similar to *hab*^*rk3*^ ^47^. However, the lack of apparent changes in adhesion molecules (E-cadherin and β-catenin) or tight junction proteins indicates that Appb facilitated adhesion might be mediated through other pathways of which the molecular mechanism needs further analysis. Nonetheless, the cell reaggregation experiment support an implication of Appb is in cell-cell adhesion. These findings challenge previous results that did not reveal any difference in cell-cell adhesion between mouse embryonic fibroblasts from *App*−/− and wild-type mice^29^. This discrepancy might further highlight the advantage of our model, since the *appb* mutant phenotype observed here likely appears before cells adapt to the loss of Appb. Thus, our data support the literature suggesting that App may act as a cell adhesion molecule not only at the synapse^29,48–50^. This is to our knowledge the first time App has been shown to play an important role in cell-cell adhesion during early development.

Cell protrusions and EVL arrangements into rosette-like structures were characteristic for *appb* mutants. The EVL is subjected to stress during epiboly and to reduce tension anisotropy, the EVL normally undergoes oriented cell divisions^27^. However, although the larger size of EVL cells may reflect an increased epithelial thinning, our data did not show any significant difference in EVL cell number or proliferation. Thus, the lack of cell loss in the EVL made us hypothesize that the rosette-like structures observed in *appb* mutants might form as constriction points around EVL gaps arising from protruding cells.

An alternative explanation would be that the weak cell-cell adhesion between EVL cells force them to rearrange to release tissue tension. Rosette-like formations are transient structures formed by epithelial cells in various tissues including the lateral line primordium, pancreas, neuronal stem cells and epithelium and retina in *Drosophila*^51^. Such rosettes are often intermediate states during cell rearrangements and can be of two types, apical or planar polarized constrictions. In the EVL, cells are conformed in a planar polarized constriction, similar to that present during tissue elongation of the *Drosophila* epithelium^52^. However, further studies are needed to elucidate the cause behind rosette formation.

The fact that most embryos with blastula defects managed gastrulation without major defects was surprising. In mice, the mild phenotype of APP−/− has been explained by redundancy with APLP2. Our results show that the loss of Appb correlates with upregulated expression of Appa. From the present study we cannot conclude on a change in Aplp2 expression since the data is conflicting. If this can be explain by the spatial resolution of whole mount *in situ* hybridization was lost in the qPCR analysis needs further evaluation. However, it is thus likely that at least Appa compensates for the loss of Appb to support normal development after gastrulation. In mice, compensatory effects have been suggested since combined mutations in APP family member genes result in increased severity of the phenotypes. However, reports on changed expression of APLPs at RNA or protein level are conflicting with some reporting no change in the APP−/−mice^10,12–14^, while others show upregulation of both APLP1 and APLP2 in APP−/− mouse brain at 8 months of age but not earlier^29^.

Recently, studies showed that mutated mRNA, especially if degraded as here, may bind and activate other genes^53–55^. Interestingly, the degraded mRNA may preferably bind and upregulate closely related genes, which could explain many of the phenotypic discrepancies observed between knockdowns (using morpholino-injected embryos) and knockouts (genetic mutation) targeting the same gene. This mechanism was found activated not only in zebrafish, but also in fly and mouse^55^. It is thus likely that degradation of mutated *appb* mRNA upregulates at least *appa*, which consequently may diminish the more severe phenotypes observed in *appb* morphant compared with *appb* knockout fish^16,17,47^. However, further studies are required to unveil the underlying mechanism behind the discrepancy in phenotypes between morphants and mutants.

Modulation of APP expression results in cellular changes that may be revealed as functional changes in locomotor behavior. In mice, loss of App leads to reduced locomotor activity^13,56^, whereas APP overexpression results in hyperactive behavior in adult mice^57^. In contrast, *appb* mutant zebrafish embryos showed no significant change in behavior at 6 dpf. This discrepancy could be explained by the fact that rodent behavioral studies are performed on adults while the zebrafish behavioral responses in this study were monitored during early development. However, we cannot exclude that compensatory mechanisms of Appa may contribute to a normal behavior as well.

In summary, we conclude that Appb ensures proper cell-cell adhesion at least during early development. This is, to our knowledge, the first time APP or its orthologues has been implicated in cell adhesion processes *in vivo*. Our data shows that while loss of Appb is partly lethal, other App family members may create a back-up system to compensate for any loss of function. How these results translate to cell interactions and homeostasis in adult zebrafish and to APP function in mammals remain a subject of study. However, as mechanisms regulating cell adhesion during development often are involved in maintaining tissue organization and homeostasis in adults^58^, it is likely that the physiological function of Appb in adhesion is maintained throughout life.

## Supplementary information

Supplementary data.

## Data Availability

Description on image analysis of cell aggregation analysis will be available at https://github.com/CamachoDejay/.
