# Supplementary material for: Amyloid precursor protein-b facilitates cell adhesion during early development in zebrafish
Source: Sci Rep. 2020 Jun 23;10:10127. doi: 10.1038/s41598-020-66584-8 (PMC7311384; doi:10.1038/s41598-020-66584-8)
Supplement: Supplementary file 1 — Supplementary data. [file 41598_2020_66584_MOESM1_ESM.pdf]

Amyloid precursor protein-b facilitates cell adhesion during early development in zebrafish

Running title: Function of Appb in zebrafish

Rakesh Kumar Banote[1#], Jasmine Chebli[1], Tuğçe Munise Şatır[1], Gaurav K. Varshney[2,3], Rafael Camacho[4], Johan Ledin[2,5] Shawn M. Burgess[2], Alexandra Abramsson[1], Henrik Zetterberg[1,6,7,8]

[1]Institute of Neuroscience and Physiology, Department of Psychiatry and Neurochemistry, The Sahlgrenska Academy, University of Gothenburg, S-41345 Gothenburg, Sweden
[2] Translational and Functional Genomics Branch, National Human Genome Research Institute, National Institutes of Health, Bethesda, Maryland 20892, USA
[3]Genes & Human Disease Program, Oklahoma Medical Research Foundation, Oklahoma City, OK, 73104, USA
[4]Centre for Cellular Imaging, Core Facilities, the Sahlgrenska Academy, University of Gothenburg, Sweden
[5]Department of Organismal Biology, Science for Life Laboratory, Uppsala University, Uppsala, Sweden
[6]Clinical Neurochemistry Laboratory, Sahlgrenska University Hospital, Mölndal, Sweden
[7]Department of Neurodegenerative Disease, UCL Institute of Neurology, Queen Square, London WC1N3BG, United Kingdom
[8]UK Dementia Research Institute, London WC1N3BG, United Kingdom

Corresponding author: alexandra.abramsson@neuro.gu.se. Institute of Neuroscience and Physiology, Department of Psychiatry and Neurochemistry, The Sahlgrenska Academy, University of Gothenburg, S-41345 Gothenburg, Sweden.
[#]Present address: Cellectricon AB, Neongatan 4B, SE-431 53, Mölndal, Sweden

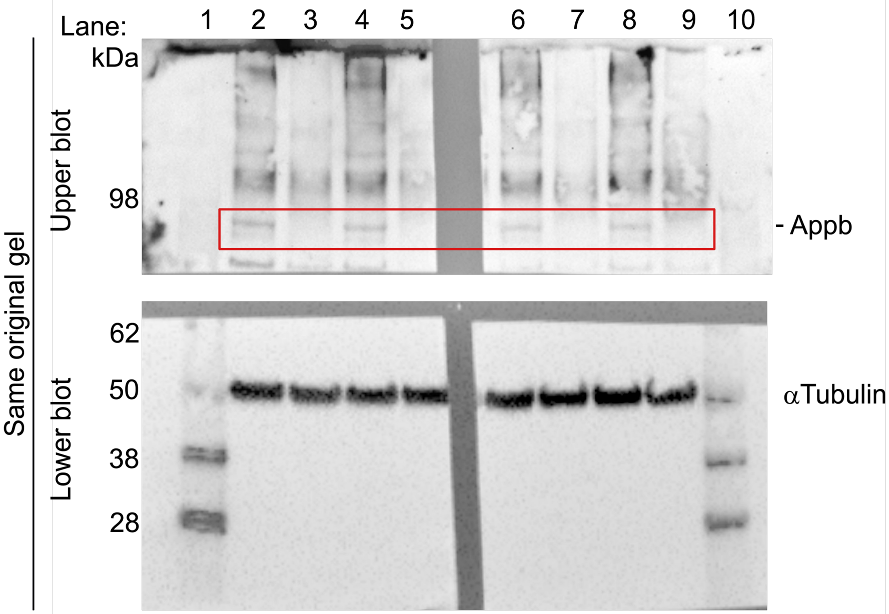

Supplementary data1D. Full size western blot analysis of Appb expression in adult brain.
Western blot with in-house generated antibody against Appb on adult brain from wild-type (n=4) and appb 26_2/26_2 mutant (n=4) zebrafish. α-tubulin was used as loading control. Upper and lower blots originate from the same gel but are cut apart and separately incubated with primary antibodies. Red box marks Appb protein bands. Lane1, See blue2 ladder, Lane2, Appb wild-type brain#1, Lane 3 Appb mut brain#1, Lane 4 Appb wild-type brain#2, Lane 5 Appb mut brain#2, Lane 6 Appb wild-type brain#3, Lane 7 Appb mut brain#3, Lane 8 Appb wild-type brain#4, Lane 9 Appb

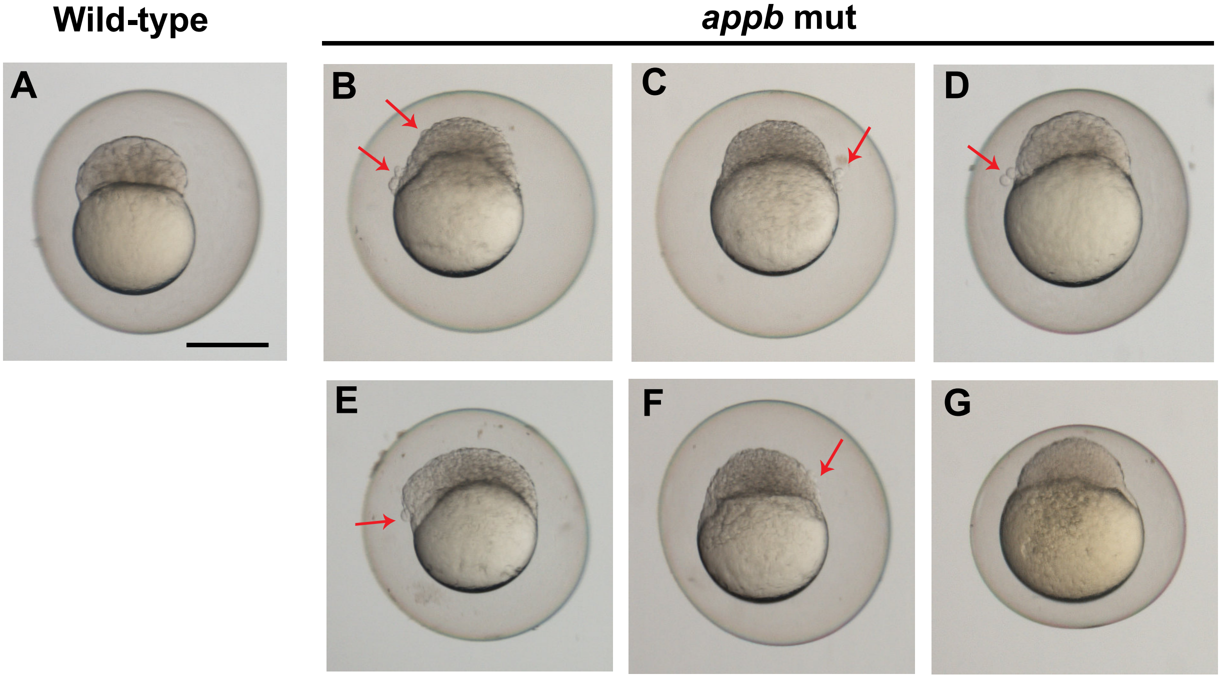

Supplementary data E. Blastula phenotypes in *appb* mutants.
Examples of *appb* wildtype (A) and *appb* mutant (B-G) blastulas displaying clusters of cells budding from the EVL (red arrows) and perched blastomeres resulting in an uneven blastula formation. Scale bar, 500µm.

Supplementary data 1F. Measurement data of embryo size

one-cell stage		
n	wild-type	*appb* mutant
1	0.75	0.70
2	0.77	0.70
3	0.75	0.70
4	0.75	0.69
5	0.75	0.70
6	0.75	0.69
7	0.76	0.68
8	0.75	0.68
9	0.73	
10	0.79	

24 hpf		
n	wild-type	*appb* mutant
1	2.01	1.94
2	1.86	1.95
3	1.88	1.88
4	1.95	1.82
5	2.12	1.82
6	2.05	1.76
7	1.93	1.75
8	2.11	1.71
9	2.15	1.81
10	2.07	1.86
11	1.87	1.82
12	2.04	1.84
13	2.10	1.82
14	1.93	1.79
15	1.89	1.80
16	1.91	1.91
17	1.94	1.96
18	2.10	1.67
19	1.97	1.94
20	2.01	1.78

48 hpf		
n	wild-type	*appb* mutant
1	3.26	3.07
2	3.42	3.25
3	3.28	3.17
4	3.42	3.22
5	3.20	3.16
6	3.35	3.13
7	3.07	3.24
8	3.35	3.16
9	3.19	3.31
10	3.39	3.17
11	3.22	2.93
12	3.38	3.09
13	3.28	3.28
14	3.19	3.09
15	3.30	3.03
16		3.12
17		3.19
18		3.11
19		3.18

3 dpf		
n	wild-type	*appb* mutant
1	3.65	3.58
2	3.75	3.71
3	3.61	3.61
4	3.70	3.71
5	3.85	3.70
6	3.66	3.51
7	3.68	3.56
8	3.58	3.77
9	3.70	3.78
10	3.80	3.74
11	3.57	3.54
12	3.85	3.68
13	3.80	3.66
14	3.65	3.66
15	3.67	3.54
16		3.63
17		3.61
18		3.59
19		3.73

Supplementary data 1G. Scoring of cell protrusion phenotype and dead embryos

Genotype	Number of embryos	Normal (number of embryos)	Cell protrusions phenotypes (number of embryos)	Dead after protrusion (number of embryos)
Wild-type	101	101	0	0
Wild-type	268	268	0	0
Wild-type	49	49	0	0
Total	418	418	0	0
%		100	0	0

Genotype	Number of embryos	Normal (number of embryos)	Cell protrusions phenotypes (number of embryos)	Dead after protrusion (number of embryos)
appb mut	79	69	10	3
appb mut	51	51	0	0
appb mut	50	37	13	6
appb mut	125	88	37	3
appb mut	46	28	18	2
appb mut	114	95	19	5
Total	465	368	97	19
%		79.14	20.86	4.09

Supplementary data 2. Analysis of Epiboly progress

	repeat 1					repeat 2					repeat 3				
	wt	wt_N	appb mut	appb mut_N	delta (h)	wt	wt_N	appb mut	appb mut_N	delta (h)	wt	wt_N	appb mut	appb mut_N	delta (h)
n	30		30			30		27			10		15		
Stage/Time (h)															
One cell	0.33	0.33	0.33	0.33	0.00	0.33	0.33	0.33	0.33	0.33	0.33	0.33	0.33	0.33	0.33
Dome	4.33	4.33	4.583	4.583	0.25	4.57	4.33	5.13	4.89	0.56	5.10	4.33	5.50	4.73	0.40
Shield	5.95	6	6.217	6.217	0.27	6.45	6.21	6.88	6.64	0.43	6.83	6.06	7.37	6.60	0.54
75%	7.833	8	8.167	8.167	0.33	8.62	8.38	9.03	8.79	0.41	9.03	8.26	9.55	8.78	0.52
100%	9.917	10	10.283	10.283	0.37	10.48	10.24	10.73	10.49	0.25	10.97	10.20	11.55	10.78	0.58
Average delay (h)					0.31					0.41					0.51

N= normalized to wild-type reaching Dome stage at 4.33h. Any delay in wild-types at this point was removed from the consecutive stages to correct for differences between repeats.

Supplementary data 4. Combined data from EVL proliferation analysis

	Genotype	Stage	No. pHH3 + EVL cells	Tot. EVL cells	pHH3+ EVL /tot EVL
1	wt	4hpf	9	117	0.077
2	wt	4hpf	11	105	0.105
3	wt	4hpf	7	131	0.053
4	wt	4hpf	12	140	0.086
5	wt	4hpf	2	133	0.015
6	wt	4hpf	10	131	0.076
7	wt	4hpf	9	171	0.053
8	wt	4hpf	8	171	0.047
1	appb mut	4hpf	6	117	0.051
2	appb mut	4hpf	10	108	0.093
3	appb mut	4hpf	7	155	0.045
4	appb mut	4hpf	3	102	0.029
5	appb mut	4hpf	15	103	0.146
6	appb mut	4hpf	6	114	0.053
7	appb mut	4hpf	12	105	0.114
8	appb mut	4hpf	17	109	0.156

Supplementary data5.
Details on Image Analysis - aggregation and cell cohesiveness assay

This document will summarize:

1. How the algorithm for aggregate segmentation works.
2. How the calculation of the parameters S and P are done from the ROI images of a segmented aggregate.1.

1. Aggregate segmentation pipeline – ROI generation:

a. Load ".nd2" file. Image data is treated as a matrix of dimensions x,y,z, where x is the number of row pixels, y is the number of column pixels and z is 2 - the number of color channels (red and green).
b. Calculate maximum intensity projection over the z dimension, effectively merging both color channels.
c. Image contrast is enhanced via the Normalize Local Contrast method - FIJI
d. Further improvement is done via a Gaussian blur – sigma 1
e. Image thresholding via Li's Minimum Cross Entropy method
f. Objects smaller than 1000 pixels are removed from the segmented image

2. Calculations of P and S for each ROI:

Calculations are based on the article: Schötz, E., Burdine, R. D., Jülicher, F., Steinberg, M. S., Heisenberg, C., & Foty, R. A. (2008). Quantitative differences in tissue surface tension influence zebrafish germ layer positioning. HFSP Journal, 2(1), 42–56. https://doi.org/10.2976/1.2834817

P - closely related to the electrical dipole moment:

Each image channel, red and green, are loaded as matrices, where the matrix value indicates intensity and the row and column index represent the pixel position. The only difference between the calculations for the red and green channel is that the pixel values of the red channel are considered negative, again in analogy to electrical charges.

For each channel, with intensity matrix $I(r,c)$, where r and c are row and column indices, respectively:

1. Image is normalized, so the total sum on the pixel intensities is 1.

$$q(r,c) = \frac{I(r,c)}{I_t}$$

$$I_t = \sum_{r,c} I(r,c)$$

2. In an analogy to the dipole moments, the moment of each pixel $\vec{\boldsymbol{P}}$ is calculated according to:

$$\vec{\boldsymbol{P}}(r,c) = q(r,c) * \vec{\boldsymbol{r}}(r,c)$$

$$\vec{\boldsymbol{r}}(r,c) = [r,c]$$

3. Then the total moment calculated by summing the moment of all pixels:

$$\vec{\boldsymbol{P}}_t = \sum_{r,c} \vec{\boldsymbol{P}}(r,c)$$

To allow for the comparison of aggregates of different size, $\vec{\boldsymbol{P}}_t$ are normalized to the radius of the aggregate. This is done by:

$$R = \sqrt{A/\pi}$$

$$\vec{\boldsymbol{P}}_n = \frac{\vec{\boldsymbol{P}}_t}{R}$$

Where, A is the area of the aggregate in units of pixels.

Finally, the parameter P is calculated by summing the red and green moments and calculating the norm of the resulting vector:

$$P = \left\| \vec{\boldsymbol{P}}_n^{green} + \vec{\boldsymbol{P}}_n^{red} \right\|$$

S - closely related to the moment of inertia and ratio of scattering amplitudes:

Each image channel, red and green, are loaded as matrices, where the matrix value indicates intensity and the row, column indices represent the pixel position.

For each channel, with intensity matrix $I(r,c)$, where r and c are row and column indices, respectively:

1. Image is normalized, so the total sum on the pixel intensities is 1.

$$I_n(r,c) = \frac{I(r,c)}{I_t}$$

$$I_t = \sum_{r,c} I(r,c)$$

2. The center of mass of the image is calculated:

$$CM_r = \sum_{r,c} I_n(r,c) * r$$

$$CM_c = \sum_{r,c} I_n(r,c) * c$$

$$\overrightarrow{\boldsymbol{CM}} = [CM_r, CM_c]$$

3. Calculate the displacement of each pixel from the center of mass:

$$\Delta r(r,c) = r - CM_r$$

$$\Delta c(r,c) = c - CM_c$$

4. Calculate the tensor of inertia according to:

$$I_{rr} = \sum_{r,c} I_n(r,c) * \left(\Delta r(r,c)\right)^2$$

$$I_{cc} = \sum_{r,c} I_n(r,c) * \left(\Delta c(r,c)\right)^2$$

$$I_{rc} = I_{cr} = \sum_{r,c} I_n(r,c) * \Delta r(r,c) * \Delta c(r,c)$$

5. Calculate the principal moments of inertia:

$$I_{max} = \frac{1}{2}\left(I_{rr} + I_{cc} + \sqrt{(I_{rr} - I_{cc})^2 + 4I_{rc}^2}\right)$$

$$I_{min} = \frac{1}{2}\left(I_{rr} + I_{cc} - \sqrt{(I_{rr} - I_{cc})^2 + 4I_{rc}^2}\right)$$

6. Calculate the scattering amplitude:

$$SA = \sqrt{\frac{I_{min} + I_{max}}{2}}$$

Finally, the parameter S is calculated as the ratio of the scattering amplitudes:

$$S = \frac{SA_{red}}{SA_{green}}$$

S is <1 if the red cells are less scattered, and >1 if the red cell are more scattered around the center of mass than the green cells. S=1 if cells are scattered equally.

Supplementary data 8.

Protein	Amino acid sequence	No.amino acids
zAplp2	KMQNHGYENPTYKYLEQMQI	20
mAplp2	KMQNHGYENPTYKYLEQMQI	20
zAppa	KMQQNGYENPTYKFFEQMHN	20
zAppB	KMQQNGYENPTYKFFEQMQN	20
mAPP	KMQQNGYENPTYKFFEQMQN	20
	::*****::***:	

Percent Identity

	zAplp2	mAplp2	zAppa	zAppb	mAPP
1: zAplp2	100.00	100.00	70.00	75.00	75.00
2: mAplp2	100.00	100.00	70.00	75.00	75.00
3: zAppa	70.00	70.00	100.00	95.00	95.00
4: zAppB	75.00	75.00	95.00	100.00	100.00
5: mAPP	75.00	75.00	95.00	100.00	100.00

Sequence alignment of the C-terminal end of App used to synthesize Y188 antibody against the mouse APP using CLUSTAL Omega. * denote concerved amino acids,: denote different in amino acids. "z" zebrafish, "m" mouse. Identity shown as percentage identity matrix.

Supplementary data 9. Locomotor activity

Total distance		High speed		Medium speed		Low speed	
Wild-type	Appb mut	Wild-type	Appb mut	Wild-type	Appb mut	Wild-type	Appb mut
71.5	11.8	21.9	0	37.7	0.6	68.2	2.7
66.7	98.1	16.6	5.7	31.4	14.6	64.1	31.9
77.9	35	2.4	11.7	22.1	23.8	50.7	52.4
80.3	62.6	25.5	9.1	36.9	17.2	69.2	38.7
56.9	88.9	1.8	58.8	2.7	61.8	4.7	103.2
52.9	0.9	9.4	28.1	20.7	43.4	43	69.9
100.2	118.7	19.3	6.6	34.3	15.5	63.4	35.9
84.4	0.1	4	23.6	11.3	39.7	22.4	59.2
73.8	53.7	15.3	3.5	34.4	13.9	70.1	31
59	101	25.2	11.7	41.6	29.8	76.5	66.2
113.7	80.9	0.5	24	2.4	29.2	6	63.1
101.4	1.1	1.3	2.1	6	9.6	13.5	26.6
43.6	30.1	17.3	4.1	33	5.3	68.5	10.4
57.1	51.5	63.2	26.4	67.1	48.1	100.6	86.2
70.2	38.8	10.9	7.5	38.3	16.8	80.2	34.5
79.6	147.1	17.8	13.7	35.5	31.6	66.2	58.2
66.9	89.7	10.4	24.1	34.3	41.6	76.1	78.2
38.6	32.6	22.4	0.1	38.6	0.6	63	1.3
82.4	78.2	21.5	44.4	39.5	48.8	70.4	90.9
5.9	26.7	9.2	0	30.2	0.1	62.9	1
42.6	62.8	10.7	12.6	25.3	26.3	56.5	53
71.5	73.2	28.4	35.7	47.6	41.5	88	81.6
21.6	19.5	20.3	17.2	40.4	42.8	81.1	74.6
71.7		24.2		32.5		60.7	
88.6		11.5		28.2		64.3	
4.6		30.3		56.9		98.3	
11		25.2		48.3		91.7	
71.4		8.2		22.7		45	
154.4		12		28.4		57.1	
71.7		17.5		31.4		68.2	
